# Hypertension in Women of Reproductive Age in the United States: NHANES 1999-2008

**DOI:** 10.1371/journal.pone.0036171

**Published:** 2012-04-30

**Authors:** Brian T. Bateman, Kate M. Shaw, Elena V. Kuklina, William M. Callaghan, Ellen W. Seely, Sonia Hernández-Díaz

**Affiliations:** 1 Division of Obstetric Anesthesia, Department of Anesthesia, Critical Care, and Pain Medicine, Massachusetts General Hospital, Harvard Medical School, Boston, Massachusetts, United States of America; 2 Division for Heart Disease and Stroke Prevention, National Center for Chronic Disease Prevention and Health Promotion, Centers for Disease Control and Prevention, Atlanta, Georgia, United States of America; 3 Division of Reproductive Health, National Center for Chronic Disease Prevention and Health Promotion, Centers for Disease Control and Prevention, Atlanta, Georgia, United States of America; 4 Department of Medicine, Brigham and Women's Hospital, Harvard Medical School, Boston, Massachusetts, United States of America; 5 Department of Epidemiology, Harvard School of Public Health, Boston, Massachusetts, United States of America; University of Florida, United States of America

## Abstract

**Objective:**

To examine the epidemiology of hypertension in women of reproductive age.

**Methods:**

Using NHANES from 1999–2008, we identified 5,521 women age 20–44 years old. Hypertension status was determined using blood pressure measurements and/or self-reported medication use.

**Results:**

The estimated prevalence of hypertension in women of reproductive age was 7.7% (95% confidence interval (CI): 6.9%–8.5%). The prevalence of anti-hypertensive pharmacologic therapy was 4.2% (95% CI 3.5%–4.9%). The prevalence of hypertension was relatively stable across the study period; the age and race adjusted odds of hypertension in 2007–2008 did not differ significantly from 1999–2000 (odds ratio 1.2, CI 0.8 to 1.7, p = 0.45). Significant independent risk factors associated with hypertension included older age, non-Hispanic black race (compared to non-Hispanic whites), diabetes mellitus, chronic kidney disease, and higher body mass index. The most commonly used antihypertensive medications included diuretics, angiotensin-converting enzyme inhibitors (ACE), and beta blockers.

**Conclusion:**

Hypertension occurs in about 8% of women of reproductive age. There are remarkable differences in the prevalence of hypertension between racial/ethnic groups. Obesity is a risk factor of particular importance in this population because it affects over 30% of young women in the U.S., is associated with more than 4 fold increased risk of hypertension, and is potentially modifiable.

## Introduction

Hypertension is a highly prevalent chronic medical condition affecting more than 65 million people in the United States [1], [2]. It is the leading reason for physician office visits, accounting for approximately 42 million ambulatory encounters each year, and is among the leading indications for the use of prescription drugs [3]. While in general, women of reproductive age have relatively low rates of hypertension, it presents important clinical implications and challenges [4] in this population, not only because of its role as a risk factor for cardiovascular disease, but also because of the issues associated with this condition and its treatment in pregnancy.

It is well established that young women with hypertension have increased risk for cardiovascular disease in both the short and the long term [5]. Rates of cardiovascular disease in young women in the U.S. appear to be increasing [6]. Analysis of U.S. vital statistics data showed that the coronary heart disease mortality rate for women age 35–44 increased on average 1.3% per year from 1997–2002; this was the only subpopulation for which the rate had increased,[6] suggesting the need for further study of risk factors in this group.

Equally important, hypertension, estimated to complicate up to 5% of the estimated 4 million pregnancies in the United States each year,[7] is a major source of maternal and fetal morbidity [7], [8]. Between 10 to 25% of women with chronic hypertension will develop superimposed preeclampsia [9], [10], [11]. The risk of placental abruption is also substantially elevated, approaching 2% in some series [9], [10]. Life-threatening maternal outcomes, including stroke [12], [13], [14], renal failure [12], [14], pulmonary edema [12], [14], and death [12], [14] are also significantly increased in women with chronic hypertension. Adverse fetal outcomes associated with chronic hypertension include preterm birth and intrauterine growth restriction [10], [11] and the perinatal mortality rate in offspring of mothers with chronic hypertension is elevated approximately 2 to 4-fold [11], [14], [15], [16].

**Table 1:** Characteristics of reproductive aged women, 20–44, United States, National Health and Nutrition Examination Survey, 1999–2008.

Characteristic	% (95% CI)^1^
Age	
20–34	57.1 (55.1–59.2)
35–39	20.7 (19.0–22.6)
40–44	22.1 (20.7–23.6)
Race/ethnicity	
White, non-Hispanic	65.5 (62.2–68.6)
Black, non-Hispanic	13.1 (11.2–15.2)
Mexican-American	9.7 (8.3–11.4)
Other	11.7 (9.7–14.1)
Diabetes^2^	2.4 (2.0–2.9)
Chronic kidney disease^3^	2.9 (2.4–3.5)
Average alcohol use^4^	
None	32.2 (29.8–34.8)
≤1 drink/day	43.8 (41.0–46.6)
>1 drink/day	24.0 (22.1–26.0)
Smoker	25.4 (23.6–27.2)
Oral contraceptive use^5^	18.6 (16.6–20.6)
Body mass index (kg/m^2^)^6^	
<25	42.4 (40.3–44.5)
25–<30	25.9 (24.3–27.7)
30–<35	16.2 (15.0–17.6)
≥35	15.5 (14.1–17.0)
Hypertension by survey cycle^7^	
1999–2000	8.0 (6.3–10.1)
2001–2002	7.3 (5.5–9.6)
2003–2004	7.2 (5.7–9.1)
2005–2006	6.0 (4.6–7.8)
2007–2008	9.3 (7.5–11.4)
Overall (1999–2008)	7.7 (6.9–8.5)

1. Weighted estimates calculated using the examination weight; 95% confidence intervals.

2. Diabetes was defined using self-reported diabetes.

3. Chronic kidney disease was defined using self-reported disease or a glomerular filtration rate of 15–60 mL/min per 1.73 m^2^.

4. Average number of drinks per day over the past year.

5. Self-reported oral contraceptive use.

6. Height and weight were measured during the examination.

7. Hypertension was defined as an average systolic blood pressure ≥140 mmHg, average diastolic blood pressure ≥90 mmHg, or self-reported currently taking anti-hypertensives.

Understanding the epidemiology of hypertension in young women may help clinicians identify important modifiable risk factors and public health officials target interventions, which in turn may improve pregnancy outcomes and prevent cardiovascular disease. There are no recent nationwide data focusing on the epidemiology of hypertension in this important group. The purpose of this study is (1) to examine prevalence of hypertension in women of reproductive age, (2) to identify factors independently associated with hypertension in this group, and (3) to analyze the medications used to treat hypertension in this population utilizing data from the National Health and Nutrition Examination Survey (NHANES) 1999–2008.

## Methods

NHANES is a nationally representative cross-sectional survey designed to assess the health and nutritional status of the U.S. civilian, non-institutionalized population. NHANES became a continuous survey in 1999; data are released in 2-year cycles. All NHANES surveys include a personal interview in the household and a detailed physical examination in a mobile examination center (MEC). Additional data on the survey design, questionnaires, and laboratory methods are available elsewhere. (Centers for Disease Control and Prevention, National Center for Health Statistics. National Health and Nutrition Examination Survey. Available at: http://www.cdc.gov/nchs/nhanes.htm. Accessed May 9, 2011.).

To reliably estimate prevalence of hypertension and anti-hypertensive medication use among women of reproductive age, data were analyzed from 5 survey periods collected from 1999 to 2008. The overall examination survey response rates ranged from 75% to 80%. During 1999–2008, 5,909 women aged 20–44 participated in the household and MEC examination. Among those, 5,521 participants had complete data to determine hypertension status and medication usage.

Hypertension was defined using blood pressure measurements and/or self-reported anti-hypertensive use. Blood pressure was measured by a physician using an appropriately sized cuff. Volunteers rested at least 5 minutes before the blood pressure readings were obtained. The average of up to 3 blood pressure measurements, obtained during the MEC examination, was used to assess blood pressure. Participants with an average systolic blood pressure ≥140 mmHg and/or an average diastolic blood pressure ≥90 mmHg or those who self-reported currently taking prescribed medication for high blood pressure were defined as hypertensive [17].

Prescription medication for respondents was obtained from the prescription medication section of the household interview. Participants were asked whether they had taken any prescription medications in the previous 30 days. Interviewers recorded prescriptions using their medication bottles. Medications recorded by NHANES are coded and classified using the Lexicon Plus® database. (Cerner, Multum, Inc. Available at: http://www.multum.com/Lexicon.htm. Accessed: May 9, 2011.) Anti-hypertensives were defined as angiotensin converting enzyme (ACE) inhibitors, angiotensin II receptor antagonists (ARBs), antiadrenergic agents, beta-blockers, calcium channel blockers, and diuretics. Combination drugs were re-categorized into the single medication classes for each of the constituents of the combination (i.e., each constituent was counted towards the total number exposed for the class of the components). For those taking more than one medication, each medication was separately counted towards the total for each class. Prescription medication was only examined among those who self-reported taking anti-hypertensives.

For each participant, potential risk factors for hypertension were abstracted from the dataset. These included age (grouped into 20–34, 35–39, and 40–44 years old) and race/ethnicity (grouped into white (non-Hispanic), black (non-Hispanic), Mexican-American, and other), diabetes mellitus (defined as self-reported disease), chronic kidney disease (defined as self-reported disease or a glomerular filtration rate (GFR) of 15–60 mL/min per 1.73 m^2^, with GFR calculated as previously described [18] ), average alcohol use over the past year (grouped as none, ≤1 drink/day, >1 drink/day), active cigarette smoking, self-reported oral contraceptive use, and body mass index (BMI) (obtained from height and weight measurement during the exam using standardized techniques and equipment [19] and grouped as <25 kg/m^2^, 25-<30 kg/m^2^, 30-<35 kg/m^2^, and ≥35 kg/m^2^). The prevalence of hypertension in each of these groups and the univariate association of these variables and hypertension was determined. All variables were then entered into a logistic regression model to identify independent associations with hypertension. Logistic regression was also used to assess for changes in the prevalence of hypertension during the study period, comparing the prevalence in1999–2000 with each subsequent two-year study interval.

To account for the complex, multistage probably survey design, analyses were conducted using SAS (version 9.2) callable SUDAAN (release 10.0). Results are described as weighted prevalence and unadjusted and adjusted weighted odds ratios. Statistical significance was defined as an alpha level <0.05.

## Results

Using NHANES from 1999–2008, we identified 5,521 women age 20–44 years old from whom blood pressure measurements were obtained. The baseline characteristics of this group are shown in Table 1. About 65% of the cohort were non-Hispanic whites, 13% non-Hispanic blacks and 10% Mexican-Americans. The estimated prevalence of diabetes was 2.4% and of chronic kidney disease was 2.9%. Approximately 25% were cigarette smokers, 24% drank on average more than one alcoholic drink each day, and 19% reported using birth control pills. Only 42% were of normal weight or lower; 26% were overweight, 16% had class I obesity (BMI 30–35 kg/m^2^), and 15% had class II or III obesity (BMI≥35 kg/m^2^). The prevalence of hypertension was relatively stable across the study period allowing for the examination of the aggregate data for all 5 survey cycles. The overall estimated prevalence of hypertension was 7.7% (95% confidence interval (CI): 6.9%–8.5%).

Table 2 shows the prevalence of hypertension by demographics and comorbidities and the univariate association of each of these characteristics with hypertension. The prevalence of hypertension increased significantly with age, from 2.7% in women age 20–34 to 18.4% in women age 40–44. Non-Hispanic blacks were more than twice as likely as non-Hispanic whites to have hypertension. Other patient characteristics associated with hypertension included diabetes, chronic kidney disease, and higher BMI. Self-reported oral contraceptive (OCP) use was inversely associated in the univariate analysis; however, the association attenuated after adjusting for age since OCP use was inversely correlated with age (compared to 20–34 year olds, the odds ratio for OCP use was 0.93 for 35–39 year olds and 0.32 for 40–44 year olds). The results of a multivariate logistic regression analysis, with all variables entered into the model, are also shown in Table 2.

**Table 2:** Prevalence of hypertension^1^ and unadjusted and adjusted odds ratios (OR) for risk of hypertension by characteristics of reproductive aged women, 20–44, United States, National Health and Nutrition Examination Survey, 1999–2008.

Characteristic	Hypertension	Unadjusted OR	p-value	Adjusted OR^3^	p-value
	% (95% CI)^2^	OR (95% CI)		OR (95% CI)	
Age					
20–34	2.7 (2.1–3.4)	Referent		Referent	
35–39	10.0 (8.1–12.2)	4.0 (3.1–5.3)	<0.01	3.3 (2.1–5.2)	<0.01
40–44	18.4 (15.6–21.5)	8.2 (5.9–11.5)	<0.01	8.2 (5.0–13.3)	<0.01
Race/ethnicity					
White, non-Hispanic	6.6 (5.5–7.8)	Referent		Referent	
Black, non-Hispanic	16.6 (14.6–18.8)	2.8 (2.2–3.6)	<0.01	2.3 (1.5–3.5)	<0.01
Mexican-American	4.4 (3.3–6.0)	0.7 (0.5–1.0)	0.03	0.6 (0.3–1.0)	0.04
Other	6.4 (4.1–9.9)	1.0 (0.6–1.6)	0.92	0.9 (0.5–1.6)	0.65
Diabetes^4,6^	35.2 (27.0–44.3)	7.3 (4.9–10.9)	<0.01	3.4 (1.9–6.1)	<0.01
Chronic kidney disease^5,6^	21.6 (14.7–30.4)	3.6 (2.2–5.9)	<0.01	2.2 (1.1–4.4)	0.03
Average alcohol use (over past year)					
None	9.4 (7.7–11.4)	Referent		Referent	
≤1 drink/day	7.1 (5.6–8.9)	0.7 (0.5–1.0)	0.09	1.1 (0.7–1.7)	0.84
>1 drink/day	7.1 (5.7–8.8)	0.7 (0.5–1.0)	0.07	0.9 (0.5–1.3)	0.48
Smoker^6^	8.9 (7.0–11.3)	1.3 (0.9–1.7)	0.13	1.0 (0.6–1.4)	0.84
Oral contraceptive use^6^	2.3 (1.3–4.2)	0.3 (0.1–0.5)	<0.01	0.6 (0.3–1.1)	0.08
Body mass index (kg/m^2^)					
<25	3.5 (2.5–4.7)	Referent		Referent	
25–<30	6.2 (4.7–8.1)	1.8 (1.2–2.9)	<0.01	2.0 (1.1–3.5)	0.03
30–<35	9.8 (7.8–12.3)	3.0 (2.0–4.6)	<0.01	4.2 (2.4–7.2)	<0.01
≥35	18.9 (16.5–21.6)	6.5 (4.4–9.6)	<0.01	6.1 (3.4–10.9)	<0.01

1. Hypertension was defined as an average systolic blood pressure ≥140 mmHg, average diastolic blood pressure ≥90 mmHg, or self-reported currently taking anti-hypertensives.

2. Weighted estimates calculated using the examination weight; 95% confidence intervals.

3. Adjusted for all variables in the table.

4. Self-reported diabetes.

5. Self-reported disease or a glomerular filtration rate of 15–60 mL/min per 1.73 m^2^.

6. Yes versus no.

**Figure 1:**
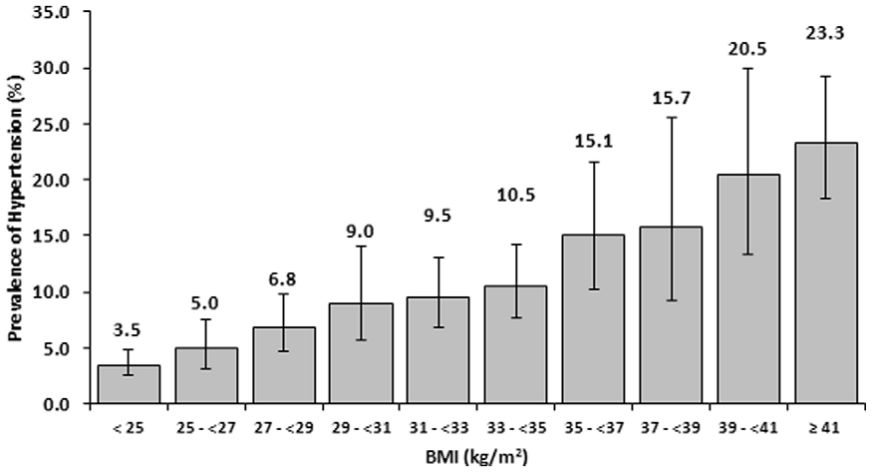
Prevalence^1^ of hypertension^2^ by body mass index (BMI)and for reproductive aged women, 20–44, United States, National Health and Nutrition Examination Survey, 1999–2008 . 1. Weighted estimates calculated using the examination weight and 95% confidence intervals. 2. Hypertension was defined as an average systolic blood pressure ≥140 mmHg, average diastolic blood pressure ≥90 mmHg, or self-reported currently taking anti-hypertensives.

**Figure 2:**
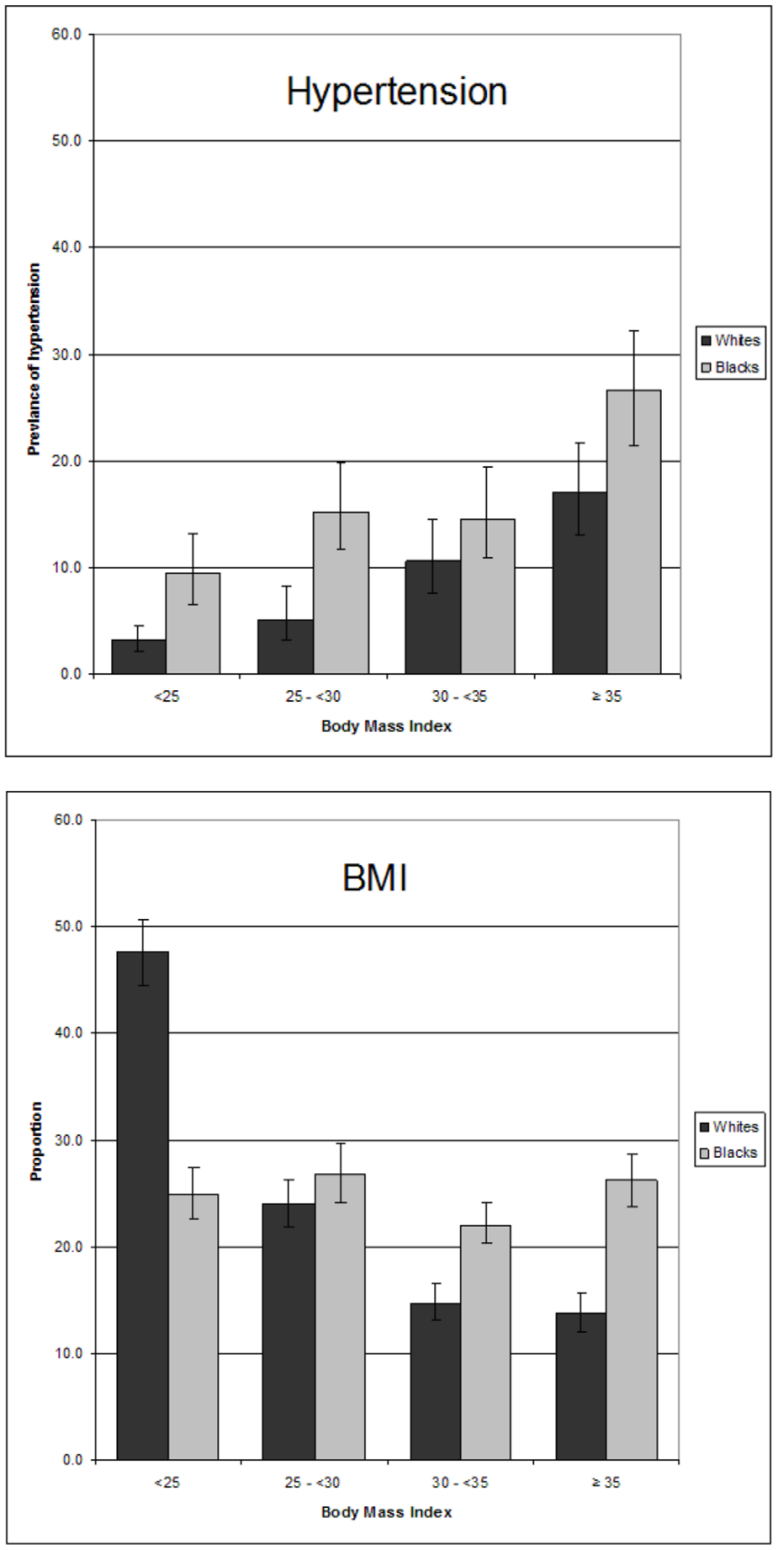
Prevalence^1^ of hypertension^2^ (A) by body mass index (B) and distribution of BMI for white and black, non-Hispanic reproductive aged women, 20–44, United States, National Health and Nutrition Examination Survey, 1999–2008. 1. Weighted estimates calculated using the examination weight; 95% confidence intervals. 2. Hypertension was defined as an average systolic blood pressure ≥140 mmHg, average diastolic blood pressure ≥90 mmHg, or self-reported currently taking anti-hypertensives.

Because increasing BMI was the most significant modifiable risk factor identified in our analysis, we explored the relationship of BMI and hypertension in more detail(Figure 1). There was a near linear increase in the prevalence of hypertension with rising BMI from 25. As shown in Figure 2, more than a quarter of all Black, non-Hispanic women had stage II or greater obesity. Blacks had higher prevalence of hypertension at every BMI compared with whites; blacks with a BMI greater than or equal to 35 kg/m^2^ had a prevalence of 26.5%.

In this study, 4.2% (95% CI 3.5%–4.9%) of women of reproductive age used anti-hypertensive pharmacologic therapy. Table 3 shows the distribution of the anti-hypertensive used by patients reporting treatment for hypertension. Among anti-hypertensive users, the most commonly used medication classes included diuretics (47.9%), ACEIs (44.0%), and beta-blockers (23.3%). For women taking ACEIs and/or ARBs, the prevalence of diabetes was 19.2% (95% CI 13.0–27.4).

**Table 3:** Anti-hypertensive use^1^ of reproductive aged women, 20–44, United States, National Health and Nutrition Examination Survey, 1999–2008.

Medication class^2^	% (95% CI)^3^
Angiotensin-converting enzyme inhibitor	44.0 (36.1–52.3)
Angiotensin receptor blockers	20.4 (13.8–29.1)
Beta blockers^4^	23.3 (16.9–31.3)
Calcium channel blockers	20.3 (15.3–26.5)
Diuretics	47.9 (39.9–55.9)

1. Medications were examined among participants who reported taking prescription anti-hypertensives within the past month and the medication container was seen by the interviewer.

2. Categories not mutually exclusive. Combination drugs were reclassified into individual medication classes. The estimate for antiadrenergic agents is not reportable because the relative standard error exceeds 30%.

3. Weighted estimates calculated using the examination weight among participants taking anti-hypertensives; 95% confidence intervals.

4. Including labetolol.

## Discussion

This study uses data from the NHANES 1999 to 2008 sample to define the prevalence and risk factors for hypertension for women of reproductive age in the United States, and to describe the relative prevalence of the medications used to treat hypertension in this group. We report an overall hypertension prevalence of 7.7%, which was relatively stable across the 10-year study period. Advancing age, non-Hispanic black race/ethnicity, diabetes, chronic kidney disease, and obesity were independently associated with hypertension in this population. An estimated 4.9% of women of reproductive age used antihypertensive pharmacologic therapy. Among anti-hypertensive users, the most common medication classes included diuretics (47.9%), ACE inhibitors (44.0%), and beta blockers (23.3%).

The most significant modifiable risk factor for hypertension that we identified in our analysis was obesity. After adjustment for other variables, women with class I obesity were approximately 4-fold and women with class II/III obesity approximately 6-fold more likely to be hypertensive than their normal weight counterparts. As shown in Figure 1, we also observed that the prevalence of hypertension increased in a near linear fashion with BMI and only started to plateau as BMI approached 40. The rising prevalence of obesity in pregnancy,[20] suggests obstetricians will be increasingly confronted with the issues of hypertension.

Non-Hispanic Black race/ethnicity and advancing age were non-modifiable patient characteristics associated with increased risk for hypertension. Multiple studies in the general population have demonstrated that hypertension in blacks is more prevalent, earlier in onset, and more severe [21], [22]. Hypertension, in part, contributes to the large disparities between white and blacks in the US in rates of cardiovascular disease [23] and adverse pregnancy outcomes [24]. Developing preventive measures for hypertension aimed at this group may be one mechanism to help decrease these disparities. It should be noted that nearly one-half of young black women were obese in this sample; preventative measures might consider targeting obesity in this population.

The increased prevalence of hypertension with advanced age, likewise, may explain some of the increased risk for some pregnancy complications in women of advanced maternal age. The problem of chronic hypertension in pregnancy is likely to become more common as the numbers of mothers of advanced age increases [25].

Approximately 5% of women of reproductive age took antihypertensive medications. Most common among these were diuretics, ACE inhibitors, and beta blockers. Recent data regarding the risks of congenital malformations associated with antihypertensive exposure during the first trimester have been mixed, with some studies reporting increased risk while others suggest that any observed risk is attributable to the underlying hypertension (“confounding by indication”) [26], [27], [28], [29], [30], [31], [32]. The Food and Drug Administration currently categorizes most antihypertensives as category C–meaning that animal studies either show an adverse effect or are lacking and no well-controlled human studies exist, and that medication should only be given when the benefit justifies the potential risk to the fetus [33]. As about half of all pregnancies in the United States are unintended [34], medications prescribed to women of reproductive age are likely to be frequently taken during the first trimester. Given the high prevalence of antihypertensive medication utilization in women of reproductive age, further research into the safety of these medications in pregnancy is merited to inform the selection of the safest antihypertensive for this population.

We found a relatively stable rate of hypertension across the study period. Data suggest that the rate of obesity in the U.S. has begun to plateau [19]. As shown in our study, obesity is an extremely important risk factor for hypertension in this population, and the lack of rise in obesity rates may explain the lack of rise in the prevalence of hypertension.

Results reported in this study should be interpreted with the following limitations in mind. First, there are several patient characteristics that are known to be associated with hypertension from previous studies, including heavy alcohol use [35], OCPs use [36], [37], [38], and cigarette smoking [39], which were not significant in our analysis of the NHANES sample. It may be that the effect of alcohol and smoking in contributing to hypertension occurs only after many years of exposure and thus the association is less robust in young women, such as those considered in our study or that some women with hypertension avoid tobacco and alcohol. In this study, OCP use in the univariate analysis was protective; however, OCP use was inversely correlated with advancing age, and after adjustment for this and other patient characteristics, OCP use was not significantly associated with hypertension. It is also likely that clinicians are reluctant to prescribe OCP to hypertensive women or discontinue OCPs if women develop hypertension, which would lead to a lack of association or even an inverse association. An additional limitation is that the lower age limit considered is 20 (as certain variables of interest are not reported in the NHANES for younger women). Likewise, in keeping with most epidemiologic studies of women of reproductive age, we defined the upper age limit for our population at 44–but women older than this can become pregnant through assisted reproduction, and hypertension is likely even more prevalent in this group. We were not able to analyze the effect of physical activity on the risk of hypertension, as the questions used to ascertain activity changed during the study period. Finally, NHANES is a cross-sectional study and it is appropriate for describing prevalence of conditions and associations, but not temporal relationships. As with any observational study, it has a limited role in establishing causality.

In conclusion, hypertension occurs in about 8% of women of reproductive age. Obesity is a risk factor of particular importance in this population because it affects over 30% of young women in the U.S., is associated with more than a 4 fold increased risk of hypertension, and is potentially modifiable. There are also remarkable differences in the prevalence of hypertension between racial/ethnic groups. Women of reproductive age are commonly exposed to antihypertensive medications and data regarding the fetal risks associated with first trimester exposure are conflicting; as a large proportion of pregnancies are unplanned, further work is needed to define the safest antihypertensive medications for these patients.
